# Gut Microbiota Dysbiosis Is Associated with Altered Bile Acid Metabolism in Infantile Cholestasis

**DOI:** 10.1128/mSystems.00463-19

**Published:** 2019-12-17

**Authors:** Yizhong Wang, Xuefeng Gao, Xinyue Zhang, Yongmei Xiao, Jiandong Huang, Dongbao Yu, Xiaolu Li, Hui Hu, Ting Ge, Dan Li, Ting Zhang

**Affiliations:** a Department of Gastroenterology, Hepatology and Nutrition, Shanghai Children’s Hospital, Shanghai Jiao Tong University, Shanghai, China; b Department of Gastroenterology and Hepatology, Shenzhen University General Hospital, Shenzhen, Guangdong, China; c Shenzhen University Clinical Medical Academy, Shenzhen, Guangdong, China; d Shenzhen HRK Bio-Tech Co., Ltd., Shenzhen, Guangdong, China; University of California, San Diego

**Keywords:** bile acids, cholestatic jaundice, dysbiosis, gut microbiota, infants

## Abstract

The co-occurrence of gut microbiota dysbiosis and bile acid (BA) metabolism alteration has been reported in several human liver diseases. However, the gut microbiota dysbiosis in infantile cholestatic jaundice (CJ) and the linkage between gut bacterial changes and alterations of BA metabolism have not been determined. To address this question, we performed 16S rRNA gene sequencing to determine the alterations in the gut microbiota of infants with CJ, and assessed their association with the fecal levels of primary and secondary BAs. Our data reveal that CJ infants show marked declines in the fecal levels of primary BAs and most secondary BAs. A decreased ratio of cholic acid (CA)/chenodeoxycholic acid (CDCA) in infants with CJ indicated a shift in BA synthesis from the primary pathway to the alternative BA synthesis pathway. The bacterial taxa enriched in infants with CJ corresponded to the genera *Clostridium*, *Gemella*, *Streptococcus*, and *Veillonella* and the family *Enterobacteriaceae* and were negatively correlated with the fecal BA level and the CDCA/CA ratio but positively correlated with the serological indexes of impaired liver function. An increased ratio of deoxycholic acid (DCA)/CA was observed in a proportion of infants with CJ. The bacteria depleted in infants with CJ, including *Bifidobacterium* and Faecalibacterium prausnitzii, were positively and negatively correlated with the fecal levels of BAs and the serological markers of impaired liver function, respectively. In conclusion, the reduced concentration of BAs in the gut of infants with CJ is correlated with gut microbiota dysbiosis. The altered gut microbiota of infants with CJ likely upregulates the conversion from primary to secondary BAs.

**IMPORTANCE** Liver health, fecal bile acid (BA) concentrations, and gut microbiota composition are closely connected. BAs and the microbiome influence each other in the gut, where bacteria modify the BA profile, while intestinal BAs regulate the growth of commensal bacteria, maintain the barrier integrity, and modulate the immune system. Previous studies have found that the co-occurrence of gut microbiota dysbiosis and BA metabolism alteration is present in many human liver diseases. Our study is the first to assess the gut microbiota composition in infantile cholestatic jaundice (CJ) and elucidate the linkage between gut bacterial changes and alterations of BA metabolism. We observed reduced levels of primary BAs and most secondary BAs in infants with CJ. The reduced concentration of fecal BAs in infantile CJ was associated with the overgrowth of gut bacteria with a pathogenic potential and the depletion of those with a potential benefit. The altered gut microbiota of infants with CJ likely upregulates the conversion from primary to secondary BAs. Our study provides a new perspective on potential targets for gut microbiota intervention directed at the management of infantile CJ.

## Abstract (precis)

Liver health, fecal bile acid (BA) concentrations, and gut microbiota composition are closely connected. BAs and the microbiome influence each other in the gut, where bacteria modify the BA profile, while intestinal BAs regulate the growth of commensal bacteria, maintain the barrier integrity, and modulate the immune system. Previous studies have found that the co-occurrence of gut microbiota dysbiosis and BA metabolism alteration is present in many human liver diseases. Our study is the first to assess the gut microbiota composition in infantile cholestatic jaundice (CJ) and elucidate the linkage between gut bacterial changes and alterations of BA metabolism. We observed reduced levels of primary BAs and most secondary BAs in infants with CJ. The reduced concentration of fecal BAs in infantile CJ was associated with the overgrowth of gut bacteria with a pathogenic potential and the depletion of those with a potential benefit. The altered gut microbiota of infants with CJ likely upregulates the conversion from primary to secondary BAs. Our study provides a new perspective on potential targets for gut microbiota intervention directed at the management of infantile CJ.

## INTRODUCTION

Infantile cholestatic jaundice (CJ) is defined as jaundice caused by elevated conjugated bilirubin in infancy (1). The incidence of CJ is about 1 in 2,500 term infants (2). Infantile CJ is uncommon but potentially indicates severe liver disease (3). It was shown that several life-threatening disorders may have cholestasis as a major presenting sign of underlying neonatal liver disease (4). The early detection of CJ and accurate diagnosis are very important for optimal treatment and a favorable prognosis (3). The etiologies of CJ are complex, including biliary obstruction/structural abnormalities and genetic/metabolic disorders (3, 4). The major biliary obstruction/structural abnormality causes of CJ in infancy are biliary atresia, duct gallstones, and choledochal cyst (3, 4). Genetic/metabolic disorder causes of CJ include alpha-1 antitrypsin deficiency, galactosemia, hypopituitarism, tyrosinemia, progressive familial intrahepatic cholestasis, cystic fibrosis, and panhypopituitarism (3). Additional causes of CJ are neonatal hepatitis, viral infection, and parenteral nutrition-associated liver disease (3, 5).

The common clinical feature of CJ is cholestasis, which is defined as reduced bile formation or flow, resulting in the retention of substances normally excreted into bile (1). Bile is produced by the liver and stored and concentrated in the gallbladder. The main components of bile are water, electrolytes, and organic molecules, including bile acids (BAs), cholesterol, phospholipids, and bilirubin (6). The BAs in bile are derived from the catabolism of highly insoluble cholesterol, which facilitates the digestion and absorption of dietary lipids and fat-soluble vitamins in the small intestine (7). The primary BAs, cholic acid (CA) and chenodeoxycholic acid (CDCA), are synthesized in the liver, stored in the gallbladder, and discharged into the small intestine after eating and play important physiological functions in food digestion and waste product elimination (6). The majority (about 95%) of primary BAs are efficiently reabsorbed from the terminal ileum (8). The remainder (about 5%) reach the colon, where they are deconjugated, dehydrogenated, and dehydroxylated by the intestinal bacteria to form secondary BAs and passively absorbed into the portal circulation (9). Once they return to the liver, the BAs are reconjugated and then resecreted together with newly synthesized bile salts. This overall process constitutes one cycle of the enterohepatic circulation. BAs have multiple endocrine functions as regulators of hepatic glucose and lipid metabolism, liver regeneration, inflammation, and the gut microbiota (7, 10). Cholestasis results in the abnormal accumulation of bile salts, bilirubin, and lipids in liver and the blood, impairing bile-mediated physiological functions (5).

The gut microbiota is a large and diverse community of microorganisms that contribute to human health and disease (11). The human gut microbiota facilitates harvesting of nutrients and energy from the ingested food and produces numerous metabolites to regulate host metabolism and immune functions (11). The gut microbiota plays a central role in the metabolism of BAs by regulating deconjugation, dehydroxylation, dehydrogenation, and epimerization to convert primary BAs into unconjugated secondary and free BAs (12). It has been shown that the gut microbiota modulates BA synthesis by regulating the expression of enzymes for BA formation (13). BA deconjugation is catalyzed by bacteria with bile salt hydrolase activity (14). Deconjugated primary BAs that escape reuptake in the small intestine are then deconjugated and dehydroxylated into secondary BAs by the 7α-dehydroxylating microbiome (15). Conversely, BAs also act as inhibitors of the gut microbiome via antimicrobial effects on gut microbes by damaging bacterial membranes and altering the intracellular macromolecular structure (14) and via indirect effects through the activation of innate immune genes in the small intestine, such as nuclear farnesoid X receptor (FXR)-induced antimicrobial peptides (16).

The co-occurrence of gut microbiota dysbiosis and BA metabolism alteration has been shown in many human liver diseases, including nonalcoholic fatty liver diseases (17, 18), primary biliary cholangitis (19), primary sclerosing cholangitis (20), and liver cholestasis (21–25), but data on pediatric populations are lacking. In the current study, we aimed to assess the mutual influences between the altered gut microbial community and BA metabolism in infantile CJ.

## RESULTS

### Clinical characteristics of infants with CJ.

As shown in Table 1, of those 56 infants with CJ enrolled in the study, 34 (34/56, 60.7%) were boys. The median age at diagnosis was 67 days (interquartile range [IQR], 55.0, 93.5 days). Fourteen infants (14/56, 25.0%) were diagnosed with biliary atresia, and eight infants (8/56, 14.3%) were confirmed to carry a gene mutation reported to cause CJ (3). There was no statistically significant difference in gender, age, and feeding type among infants with CJ and impaired hepatic function (IHF) and healthy control (HC) infants. Blood tests showed elevated levels of BA (158.5 μmol/liter; IQR, 115.3, 213.5 μmol/liter), direct bilirubin (DB; 93.0 μmol/liter; IQR, 70.5, 123.7 μmol/liter), total bilirubin (TB; 163.1 μmol/liter; IQR, 120.2, 214.2 μmol/liter), and gamma-glutamyltransferase (GGT; 149.0 U/liter; IQR, 99.5, 316.8 U/liter) in infants with CJ, and these were significantly higher than those in infants with IHF (Table 1). High levels of alanine aminotransferase (ALT; 214.0 U/liter; IQR, 127.3, 349.0 U/liter) and aspartate aminotransferase (AST; 121.0 U/liter; IQR, 76.5, 233.8 U/liter) were also observed in infants with CJ, and the ALT level was significantly higher in infants with CJ than in subjects with IHF. Additionally, slightly lower levels of albumin (ALB) and hemoglobin (HB) were detected in infants with CJ than in subjects with IHF.

**TABLE 1:** Characteristics of infants with CJ, IHF, and HC^*a*^

Characteristic	Value for the following group:			*X*^2^/*Z*^*c*^	*P*
	HC (*n* = 45)	IHF (*n* = 25)	CJ (*n* = 56)		
No. (%) of infants by gender				2.000	0.955
Boys	26 (57.8)	15 (60.0)	34 (60.7)		
Girls	19 (42.2)	10 (40.0)	22 (39.3)		
Median (IQR) age (days)	90.0 (60.0, 140.0)	79.0 (61.0, 145.0)	67.0 (55.0, 93.5)	2.650	0.080
No. (%) of infants with the following feeding type:				4.000	0.215
Breast-feeding	19 (42.2)	11 (44.0)	12 (21.4)		
Formula	7 (15.6)	5 (20.0)	13 (23.2)		
Combination	19 (42.2)	9 (36.0)	31 (55.4)		
No. (%) of infants with the following diagnosis:				3.000	0.001
CMV infection		6 (24.0)	3 (5.4)		
Genetic disorders		0 (0)	8^*b*^ (14.3)		
Biliary atresia		0 (0)	14 (25.0)		
Unknown		19 (76.0)	31 (55.3)		
Median (IQR) concn					
ALB (g/liter)		41.0 (39.0, 43.0)	39.0 (36.0, 41.3)	−2.573	0.010
ALT (U/liter)		138.0 (62.0, 201.0)	214.0 (127.3, 349.0)	−2.689	0.007
AST (U/liter)		118.0 (56.0, 188.0)	121.0 (76.5, 233.8)	−0.854	0.393
BA (μmol/liter)		33.0 (15.0, 72.8)	158.5 (115.3, 213.5)	−4.944	0.000
DB (μmol/liter)		4.0 (2.1, 7.0)	93.0 (70.5, 123.7)	−7.157	0.000
HB (g/liter)		112 (99.0, 119.0)	101.5 (93.8, 111.0)	−1.663	0.096
GGT (U/liter)		78.0 (49.0, 167.0)	149.0 (99.5, 316.8)	−2.986	0.003
TB (μmol/liter)		12.4 (7.4, 18.3)	163.1 (120.2, 214.2)	−6.748	0.000

a ALB, albumin; ALT, alanine aminotransferase; AST, aspartate aminotransferase; BA, bile acid; DB, direct bilirubin; HB, hemoglobin; GGT, gamma-glutamyltransferase; TB, total bilirubin; IQR, interquartile range; CMV, cytomegalovirus; HC, health control; IHF, impaired hepatic function; CJ, cholestatic jaundice.

b Two infants had Alagille syndrome with a Jagged 1 (JAG1) mutation, four had citrin deficiency with a solute carrier family 25 member 13 (SLC25A13) mutation, and two had BA synthesis defects with aldo-keto reductase family 1 member D1 (AKR1D1) mutation.

c The data were compared by the nonparametric Mann-Whitney test (two groups) or Kruskal-Wallis H test (multiple groups). The distribution of dichotomous variables was compared by the chi-squared test.

### Fecal BA profiles are significantly altered in infants with CJ.

**FIG 1:**
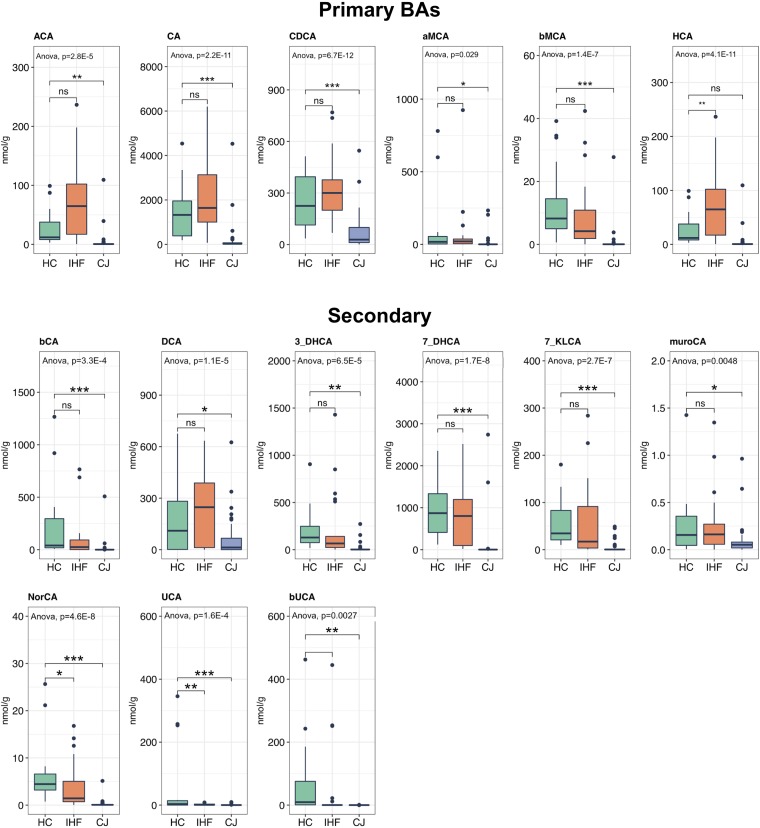
Alteration of fecal bile acid metabolism in infantile cholestasis. The levels of primary and secondary bile acids in the cohort of infants with cholestatic jaundice (CJ) or impaired hepatic function (IHF) and the health controls (HC) were measured by tandem mass spectrometry. Significant differences were determined by one-way analysis of variance (ANOVA) followed by Tukey’s *post hoc* test. ns, not significant; *, *P* < 0.05; **, *P* < 0.01; ***, *P* < 0.001. HCA, hyocholic acid; 3_DHCA, 3-dehydrocholic acid; 7_DHCA, 7-dehydrocholic acid; KLCA, ketolithocholic acid; UCA, ursocholic acid; bUCA, β-ursocholic acid; muroCA, murocholic acid; NorCA, norcholic acid.

We used a targeted metabolomics approach to evaluate the fecal BA profiles in the 95 individuals enrolled in this study, including 54 infants with CJ, 25 infants with IHF, and 16 HC infants. The levels of all primary BAs were decreased in infants with CJ, and these decreases were significant for allocholic acid (ACA), CA, CDCA, and α- and β-muricholic acid (α- and β-MCA) (Fig. 1). Reduced levels of most of the secondary BAs were found in infants with CJ, with the exceptions being α- and β-hyodeoxycholic acid (α- and β-HDCA) (Fig. 1). The patterns of decrease were also observed for the primary and secondary conjugated BAs in infants with CJ, although statistical significance was not reached. In order to evaluate the enzymatic processes in BA metabolism that may underlie the differences noted in infants with CJ, we investigated the two ratios (Table 2) reflective of enzymatic activities in the liver and the gut microbiome proposed previously (26). The CA/CDCA ratio was measured and was found to be significantly lower in infants with CJ, reflecting a shift in BA synthesis from the primary to the alternative BA pathway that occurs in the liver. The deoxycholic acid (DCA)/CA ratio was increased in infants with CJ, although the difference was not significant, indicating that the enhanced production of secondary BAs occurred in a proportion of infants with CJ.

**TABLE 2:** Ratios of BAs reflective of liver and gut microbiome and enzymatic activities^*a*^

Ratio informative about metabolic processes	Ratio calculated	Mean (95% CI)			*P* (Tukey’s test)	
		HC	IHF	CJ	HC vs IHF	HC vs CJ
BA synthesis: primary vs alternative pathway	CA/CDCA	6.745 (1.905)	6.683 (1.27)	1.818 (0.408)	1	4.2E−09
Conversion from primary to secondary BA by gut bacteria	DCA/CA	0.207 (0.159)	0.29 (0.22)	1.179 (0.636)	0.9	0.16

a BA, bile acid; CA, cholic acid; CDCA, chenodeoxycholic acid; DCA, deoxycholic acid; HC, health control; IHF, impaired hepatic function; CJ, cholestatic jaundice; CI, confidence interval.

### CJ is associated with gut microbiota dysbiosis.

**FIG 2:**
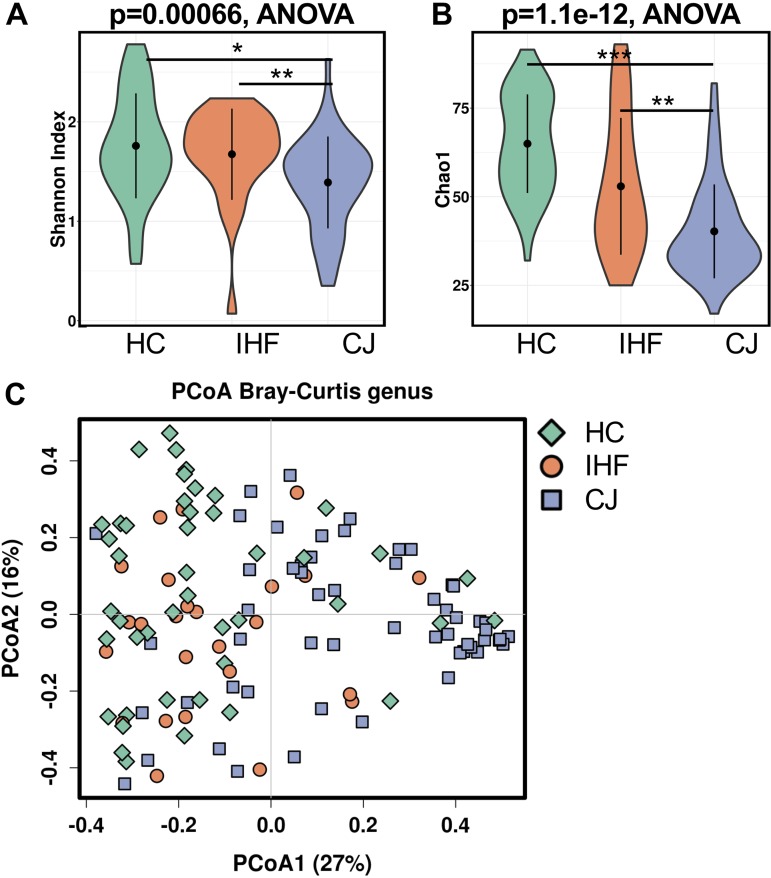
Comparison of the taxonomic diversity of the gut microbiomes among the cholestatic jaundice (CJ), impaired hepatic function (IHF), and healthy control (HC) groups. The gut microbiota alpha diversity was measured from the Shannon (A) and Chao1 (B) indexes. The Wilcoxon test was performed for pairwise comparisons. *, *P* < 0.05; **, *P* < 0.01; ***, *P* < 0.001. (C) PCoA of Bray-Curtis distances generated from taxa summarized at the genus level. Each point corresponds to a sample shaped and colored by diagnosis.

To investigate the changes in the gut microbiome, we performed 16S rRNA gene sequencing on 126 fecal samples from infants with CJ (*n* = 56), infants with IHF (*n* = 25), and HC infants (*n* = 45). Alpha diversity, measured by the Shannon and Chao1 indexes, was significantly lower in infants with CJ than those with IHF and HC (Fig. 2A and B). Principal-coordinate analysis (PCoA) of a Bray-Curtis distance matrix generated from genus-level taxa showed a separation between the microbiomes of infants with CJ and HC infants (Fig. 2C), whereas the samples from infants with IHF were not able to be distinguished from those of the infants in the other two groups.

**FIG 3:**
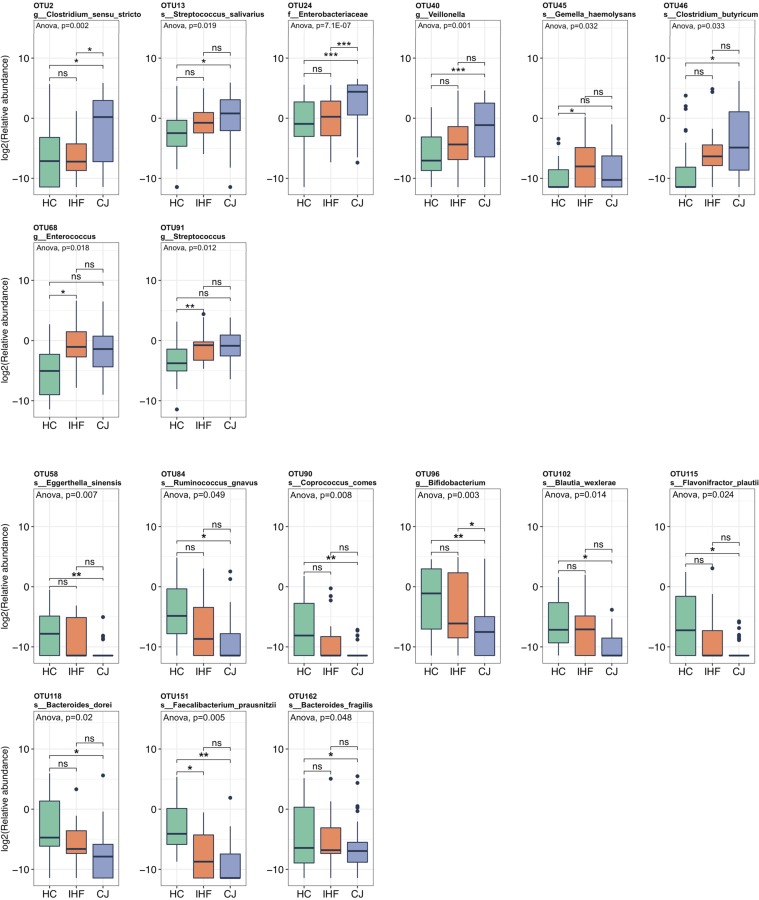
Significant differences between the gut bacterial taxa of the cholestatic jaundice (CJ), impaired hepatic function (IHF), and healthy control (HC) groups. Comparisons among the groups were performed using one-way analysis of variance (ANOVA; *P* < 0.05). The Wilcoxon test was performed for pairwise comparisons, *, *P* < 0.05; **, *P* < 0.01; ***, *P* < 0.001. g, genus; s, species; f, family.

We obtained 17 operational taxonomic units (OTUs) with a significantly different abundance among the three groups (*P < *0.05 by one-way analysis of variance [ANOVA]; these OTUs consisted of taxa that had an abundance of greater than 0.1% across all samples and that occurred in at least 20% of the samples) (Fig. 3; see Table S1 in the supplemental material). CJ patients showed increased abundances of taxa assigned to the genera *Clostridium sensu stricto* (OTU2 and OTU46), *Streptococcus* (OTU13), and *Veillonella* (OTU40) and the family *Enterobacteriaceae* (OTU24) compared with the HC. Some taxa belonging to the genus *Enterococcus* (OTU68), *Gemella* (OTU45), and *Streptococcus* (OTU91) were more abundant in infants with IHF than in HC. The levels of potentially beneficial bacteria, such as *Bifidobacterium* (OTU96) and Faecalibacterium prausnitzii (OTU151), were significantly decreased in infants with CJ. The levels of the taxon Faecalibacterium prausnitzii (OTU151) were also significantly reduced in children with IHF. In addition, the relative abundances of some taxa belonging to the genera *Bacteroides*, *Blautia*, *Coprococcus*, *Eggerthella*, and *Flavonifractor* and the *Lachnospiraceae* were also significantly reduced in infants with CJ compared with the HC.

10.1128/mSystems.00463-19.2 TABLE S1 OTUs with significantly different abundances among the cholestatic jaundice (CJ), impaired hepatic function (IHF), and healthy control (HC) groups. Download Table S1, DOCX file, 1.0 MB. Copyright © 2019 Wang et al. 2019 Wang et al. This content is distributed under the terms of the Creative Commons Attribution 4.0 International license.

### CJ-related gut microbiome functional alterations.

**FIG 4:**
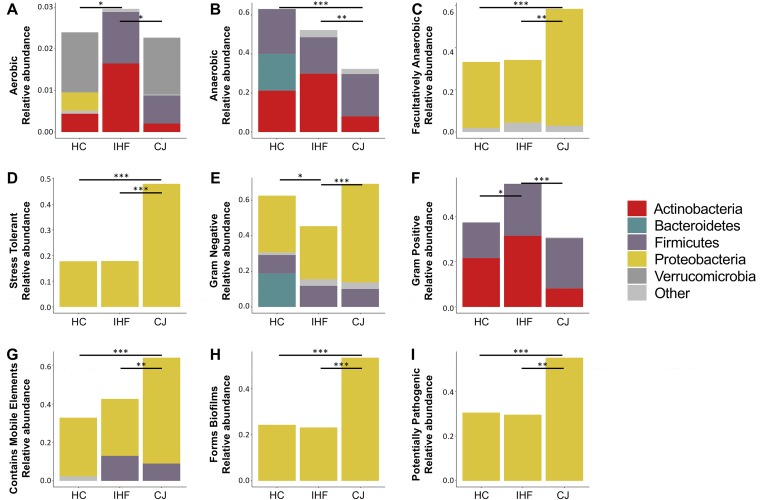
Discrepancy in microbial community phenotypes between the cholestatic jaundice (CJ) group and the other two groups. BugBase identified phenotypes associated with aerobic bacteria (A), anaerobic bacteria (B), facultatively anaerobic bacteria (C), oxidative stress tolerance (D), Gram-negative bacteria (E), Gram-positive bacteria (F), mobile element content (G), biofilm formation (H), and pathogenesis (I). Statistical significance was identified by the Wilcoxon test with false discovery rate (FDR)-corrected pairwise *P* values. *, *P* < 0.05; **, *P* < 0.01; ***, *P* < 0.001.

BugBase software was applied to analyze the community-wide phenotypes of the stool microbiome. We observed that the proportion of aerobic bacteria was increased modestly in infants with IHF (Fig. 4A). The proportions of anaerobic bacteria (Fig. 4B) were significantly reduced and those of facultatively anaerobic bacteria (Fig. 4C) were enriched in infants with CJ. Bacterial function-associated oxidative stress tolerance (Fig. 4D) was found to be enriched in the CJ subjects. Gram-negative bacteria (Fig. 4E) were decreased and Gram-positive bacteria (Fig. 4F) were greatly increased in infants with IHF in comparison with their levels in HC and CJ subjects. In addition, the bacterial function-associated mobile element content (Fig. 4G), biofilm formation (Fig. 4H), and pathogenesis (Fig. 4I) were found to the enriched in the CJ subjects. These findings were mainly attributed to the proteobacteria (most likely the *Enterobacteriaceae* family).

**FIG 5:**
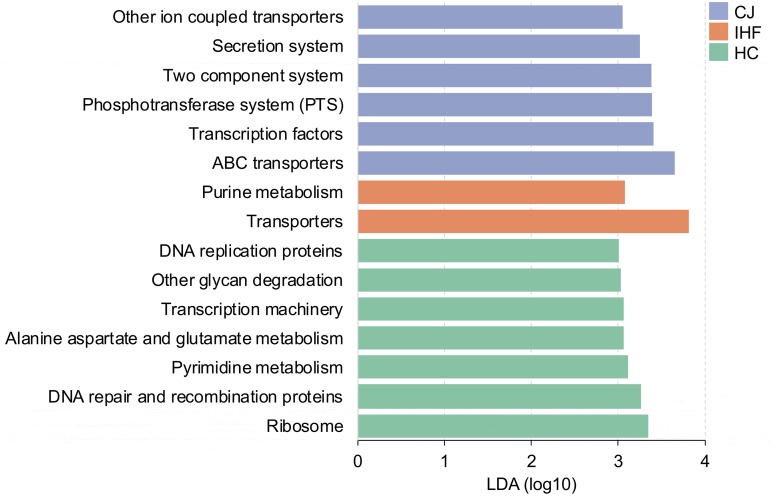
Functional alterations of the gut microbiome in infants with cholestatic jaundice (CJ) and impaired hepatic function (IHF). Statistical significance was determined by using LEfSe, with a *P* value of <0.05 (Wilcoxon test) and a linear discriminant analysis (LDA) score (log_10_) of >3 being considered significant.

PICRUSt (Phylogenetic Investigation of Communities by Reconstruction of Unobserved States) software was used to predict the relative abundance of KEGG functions, which were compared between children with CJ and IHF and the HC. We found that bacterial transporters were increased in children with altered liver function (both infants with CJ and infants with IHF) compared to the HC subjects (Fig. 5). In addition, ATP-binding cassette (ABC) transporters were particularly more abundant in CJ subjects than in the HC.

### Covariance between serum BAs, fecal BAs, and the gut microbiome.

**FIG 6:**
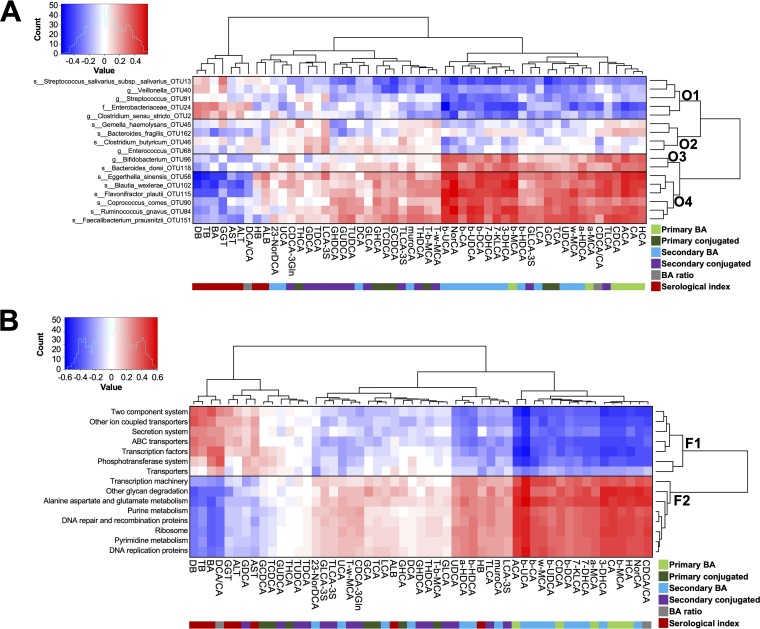
Correlation matrices constructed from gut bacterial taxa, fecal BAs, and serum indicators of hepatic function. Spearman correlation analysis between fecal BA serum indicators of hepatic function and the top 17 OTUs with significantly different abundances among the groups (A) and the gut microbiota functional categories (B) was employed. Four distinct OTU clusters (clusters O1 to O4) and two distinct functional clusters (clusters F1 and F2) were observed. Red and blue represent the positive and negative correlations, respectively. GHDCA, glycohyodeoxycholic acid; TCA, taurocholic acid; GCA, glycocholic acid; LCA, lithocholic acid; GLCA, glycolithocholic acid; THDCA, taurohyodeoxycholic acid; TLCA-3S, taurolithocholic acid-3-sulfate acid; GCDCA, glycochenodeoxycholic acid; GHCA, glycohyocholic acid; TUDCA, tauroursodeoxycholic acid; GDCA, glycodeoxycholic acid; THCA, taurohyocholic acid; TDCA, taurodeoxycholic acid; GUDCA, glycoursodeoxycholic acid; ALB, albumin; ALT, alanine aminotransferase; AST, aspartate aminotransferase; BA, bile acid; DB, direct bilirubin; HB, hemoglobin; GGT, gamma-glutamyltransferase; TB, total bilirubin.

To examine the relationship between members of the gut microbiota and BAs, we calculated Spearman’s rank correlation coefficient for the 17 OTUs (those that were significantly changed in infants with CJ and/or IHF), serological indexes, and fecal BAs. We performed unsupervised clustering of OTUs, serological parameters, and fecal BAs, which revealed four distinct OTU clusters (clusters O1 to O4; Fig. 6A) and a clear separation of fecal BAs and serological indexes. OTUs in the first cluster, cluster O1 (which included taxa assigned to the genera *Clostridium sensu stricto*, *Veillonella*, and *Streptococcus* and the family *Enterobacteriaceae*), were positively and negatively correlated with the levels of serum BAs (and most of the serological indexes) and fecal BAs, respectively. In contrast, the OTUs in cluster O4 (which included the taxa *Blautia*, *Eggerthella*, *Faecalibacterium*, *Flavonifractor*, *Lachnospiraceae incertae sedis*, and *Ruminococcus*) were negatively and positively correlated with the levels of serum BAs and fecal BAs, respectively. Notably, clusters O1 and O4 were positively and negatively correlated with the ratio of secondary/primary BAs (DCA/CA), respectively.

The associations between the functional profiles of the gut microbiota and hepatic function and fecal BAs were also investigated. Two distinct functional clusters (clusters F1 and F2) resulted from applying Spearman correlations between the PICRUSt-predicted KEGG pathways representative of each group (linear discriminant analysis [LDA] score > 3, *P* < 0.05), serological indexes, and fecal BAs (Fig. 6B). The KEGG pathways in cluster F1, which included components related to other ion-coupled transporters, the phosphotransferase system, the secretion system, transcription factors, transporters, ABC transporters, and two-component systems, were found to be positively associated with most serological indexes and inversely associated with the fecal levels of BAs, which were stronger for primary and secondary BAs than for their conjugated components. The KEGG pathways in cluster F2, which included alanine aspartate and glutamate metabolism, DNA replication proteins, DNA repair and recombination proteins, other glycan degradation, purine metabolism, pyrimidine metabolism, ribosome, and transcription machinery, were negatively and positively correlated with serum BAs (as well as other serological indexes of liver functions) and fecal BAs, respectively. The levels of KEGG pathways in cluster F1 were found to be positively and negatively correlated with DCA/CA and CDCA/CA, respectively, whereas the functions of cluster F2 showed the opposite trend of correlations.

## DISCUSSION

The primary BAs are synthesized in the liver through two pathways, namely, the classical (neutral) and the alternative (acidic) pathways. The classical BA synthetic pathway produces about 90% of total BAs, composed of CA and CDCA in approximately equal amounts. In contrast to the classical pathway, the alternative pathway produces only CDCA (6). Alterations in BA synthesis and composition are important factors that modulate hepatotoxicity in chronic liver diseases. Liver health, fecal BA concentrations, and the gut microbiota composition are closely connected (22). BAs and the microbiome influence each other in the gut, where bacteria modify the BA profile, while intestinal BAs regulate the growth of commensal bacteria, maintain barrier integrity, and modulate the immune system (22). An inborn error of BA synthesis can produce abnormal BA metabolites, which can lead to cholestatic liver disease in infancy and progressive neurological disease in childhood and into adulthood (27, 28). In this study, we demonstrated the association between the changes of the gut microbiome and BA metabolism and estimated the potential impact on host physiology in infant CJ patients. We observed reduced levels of the primary BAs and most of the secondary BAs in stool samples from infants with CJ, a finding which is consistent with the typical symptoms of cholestasis. Infants with CJ showed a decreased fecal CA/CDCA ratio, indicating that the classic pathway was impaired, while the alternative one was preserved. In addition, an increase in the ratio of secondary to primary BAs (DCA/CA) was observed in infants with CJ, which may suggest altered activity of bacterial 7α-dehydroxylases, leading to an excess production of secondary BAs, many of which are cytotoxic to the liver. Actually, the concentrations of two secondary BAs, α- and β-HDCA, were found to be increased in infants with CJ compared with the HC and infants with IHF.

We demonstrated that the CJ-associated gut microbiota dysbiosis was characterized by a decreased biodiversity, a decrease in the microbiota with a beneficial potential (the genera *Bifidobacterium* and *Faecalibacterium*), and an overgrowth of potentially pathogenic bacteria (the *Enterobacteriaceae* family). Species of the genus *Bifidobacterium* are believed to confer beneficial effects upon their host, and their high abundance in the infant gut indicates saccharolytic activity toward glycans. *Bifidobacterium* appears to influence short-chain fatty acid (SCFA) production, either directly, by modulating the synthesis of SCFAs, such as acetate and formate (29), or indirectly, by altering the gut microbiota composition and/or microbiome-microbiome interactions (30). In addition, species of the genus *Bifidobacterium* also contribute to maintaining the normal function of the gut mucosa and protect the mucosa from injurious factors, such as allergens, pathogens, and toxins (31). The genus *Faecalibacterium*, which comprises the sole known species Faecalibacterium prausnitzii, consists of well-established protective bacteria with the capacity to reinforce the barrier integrity of epidermal cells and repress inflammation through the production of SCFAs (32, 33). Thus, declines in the abundances of *Bifidobacterium* and Faecalibacterium prausnitzii may evoke and enhance systemic inflammation in the host. A member of the family *Enterobacteriaceae* was significantly enriched in the gut microbiota of CJ infants, resulting in an increased level of bacterial gene contents with pathogenic potential, and its abundance was positively correlated with serum indicators of liver damage. Additionally, lower abundances of *Lachnospiraceae* and *Blautia* were detected in infants with CJ, which is similar to the findings for patients with cirrhosis (21, 22).

The known gut bacteria capable of processing 7α-dehydroxylases are the species of *Clostridium* (such as Clostridium hiranonis, C. hylemonae, C. scindens, and C. sordellii) (15). Due to a lack of redundancy in this system, any perturbations to the 7α-dehydroxylating bacteria are likely to induce an imbalance in the ratio between secondary and primary BAs and impact hepatic function. Although two taxa of *Clostridium* were found to be enriched in the infants with CJ, their identification to the species level could not be accurately determined based on the sequences of the 16S rRNA V3-V4 regions. Thus, further investigations of the microbiome profile with shotgun metagenomic sequencing are needed to identify the exact species that shift 7α-dehydroxylation during cholestasis.

Functional analysis of the bacterial communities in the infants with CJ revealed the enhanced expression of ABC transporters. In the host system, ABC transporters serve as an important cytoprotective mechanism by eliminating toxins and drugs out of cells, thereby protecting the hepatocyte from toxicity due to BA overaccumulation. Bacterial ABC transporters are also able to protect the microorganisms from xenobiotic pressure (34). The apparent upregulation of ABC transporters and other ion-coupled transporters might implicate an enhanced antimicrobial pressure in the gut environment.

In summary, infantile CJ is associated with a significant dysbiosis of the gut microbiota, which is featured by a general decline in bacteria with beneficial potential as well as those capable of processing the necessary enzymes to convert primary to secondary BAs and an enrichment of potentially pathogenic taxa. These changes in the gut microbiota might modulate the composition of BAs, disrupt the gut barrier integrity, and provoke and enhance inflammation.

## MATERIALS AND METHODS

### Study cohort.

A total of 126 infants from Shanghai Children’s Hospital, Shanghai, China, were recruited to the study cohort between September 2016 and March 2019 (Table 1). The diagnosis of CJ in the infants was based on the guidelines described elsewhere (1, 3). An abnormal direct bilirubin (DB) level was defined as a value greater than 17 μmol/liter if the total bilirubin (TB) level was less than 85 μmol/liter or a value of DB that represented more than 20% of that of TB if the TB level was greater than 85 μmol/liter (1). Serological tests for liver function were performed. Levels of BA, DB, TB, gamma-glutamyltransferase (GGT), aspartate aminotransferase (AST), alanine aminotransferase (ALT), and albumin (ALB) were measured by using an AU5800 clinical chemistry analyzer (Beckman Coulter, Brea, CA, USA). Hemoglobin (HB) was measured by use of a sodium lauryl sulfate (SLS) hemoglobin test (Sysmex XN-1000 automatic hematology analyzer; Sysmex Corporation). Twenty-five infants with normal levels of DB and TB but with elevated AST and ALT levels were enrolled in the study as controls and were termed the impaired hepatic function (IHF) group. Forty-five healthy infants without any history of chronic diseases were enrolled as healthy controls (HC). None of the healthy individuals had clinically relevant CJ or any symptoms at the time of fecal collection, and subjects who took antibiotics within 2 months before fecal collection or who had any inflammatory conditions were excluded from the study. Written informed consent was obtained from the parents or legal guardians of the infants eligible for study enrollment. This study was approved by the Regional Ethical Review Boards of Shanghai Children’s Hospital and carried out in accordance with the principles of the Declaration of Helsinki of 1964 and later versions.

### Quantification of fecal BAs.

Targeted metabolomics profiling was performed to measure the concentrations of 43 BAs in fecal samples according to previously reported methods (35). In brief, each accurately weighed lyophilized fecal sample (∼10 mg) was homogenized with 50 μl of water using a Bullet Blender tissue homogenizer (Next Advance, Inc., Averill Park, NY). An aliquot of 150 μl of acetonitrile containing 9 internal standards was added, and the extraction was performed using the homogenizer. After centrifugation, 50 μl of each supernatant was transferred to a 96-well plate and diluted with 150 μl of a mobile phase mixture (mobile phase B-mobile phase A [50:50, vol/vol]). The injection volume was 5 μl. After centrifugation, 5 μl supernatant was used for measurement by liquid chromatography-tandem mass spectrometry analysis (LC-TQMS). An Acquity ultraperformance liquid chromatography (UPLC) system (Waters Corp., Milford, MA, USA) coupled with a Xevo TQ-S mass spectrometer (Waters Corp., Milford, MA, USA) was used to quantitate the BAs. MassLynx software (version 4.1; Waters Corp., Milford, MA, USA) was used for instrument control and data processing. Chromatographic separation was achieved with a Waters BEH C_18_ column (particle size, 1.7 μm; 2.1 mm by 100 mm [internal dimensions]). The UPLC-mass spectrometry (MS) raw data were acquired in negative mode and were processed using the TargetLynx application manager (Waters Corp., Milford, MA, USA) to obtain calibration equations and the measured concentration of each bile acid in the samples.

### Fecal microbiome analysis.

Stool samples were collected with sterile swabs by nursing staff at Shanghai Children’s Hospital and stored at −80°C. Genomic DNA extraction, PCR amplification, library preparation, and Illumina sequencing were conducted according to a protocol described previously (36). In brief, total microbial DNA was extracted using a QIAamp DNA stool minikit (Qiagen, Germany). The extracted genomic DNA was PCR amplified with barcoded primers (forward primer, 5′-CCT ACG GGA GGC AGC AG-3′; reverse primer, 5′-GGA CTA CHV GGG TWT CTA AT-3′) targeting the 16S rRNA V3-V4 region. Water samples that had undergone the same procedures of DNA extraction and PCR amplification were used as a control. An equal amount of DNA from each sample was pooled and verified using an Agilent 2100 bioanalyzer (Agilent, USA). Sequencing was performed using an Illumina MiSeq platform at HRK Bio-Tech Co., Ltd. (Shenzhen, China).

A total of 8,265,997 raw sequence reads were produced from 126 stool samples. The paired reads were merged. The average number of reads per sample was 65,603 ± 23,222. The average length after joining of the reads was 462 ± 8 bp per sample. Data processing was performed by using the USEARCH (version 10.0.240) program (37) with an open-source bioinformatics pipeline described at http://www.drive5.com/usearch. The depth of the sequencing data used for analysis was 10,000 reads per sample, based on the findings of the rarefaction analysis (see Fig. S1 in the supplemental material). We applied the Unoise error correction (denoising) algorithm to reconstruct a set of correct biological sequences in the reads and generate zero-radius operational taxonomic units (OTUs) (38). Taxonomic assignment was performed by using the Ribosomal Database Project (RDP) classifier. Statistical analysis was performed with the Calypso web server (39). The alpha diversity of the fecal microbiome was measured by use of the Shannon and Chao1 indexes. The overall differences in microbiome structure were evaluated through principal-coordinate analysis (PCoA) of a Bray-Curtis distance. BugBase was applied to predict the organism-level microbiome phenotypes (40). The KEGG ortholog functional profiles of the microbial communities were predicted by PICRUSt (Phylogenetic Investigation of Communities by Reconstruction of Unobserved States) software (41). The linear discriminant analysis (LDA) effect size method (LEfSe) was applied to determine the PICRUSt-predicted functions that were enriched in the different groups. The differences in the relative abundances of the taxa and the concentrations of BAs among the groups were assessed for statistical significance by one-way ANOVA followed by Wilcoxon pairwise comparisons. The Spearman correlation was applied to investigate the associations between serological indexes, fecal BAs, and the composition and functions of the gut microbiome.

10.1128/mSystems.00463-19.1 FIG S1 Rarefaction analysis of the 16S rRNA genes in the fecal microbiome. Rarefaction plots of all 126 samples show the number of observed OTUs reaching the asymptote at the cutoff of 10,000 reads. Each sample is represented by a different color in the graph. Download FIG S1, TIF file, 1.0 MB. Copyright © 2019 Wang et al. 2019 Wang et al. This content is distributed under the terms of the Creative Commons Attribution 4.0 International license.

### Statistical analysis.

Statistical analysis was performed with SPSS (version 20.0) software. The data are presented as medians and interquartile ranges (IQR; 25th to 75th percentiles) and compared by use of the nonparametric Mann-Whitney test (for two groups) or the Kruskal-Wallis H test (for multiple groups). The distribution of dichotomous variables was compared by the chi-squared test. A *P* value of <0.05 was considered statistically significant.

### Data availability.

Raw metabolomic data for the bile acids are available in the Metabolights metabolomics repository under study accession number MTBLS1295. Raw sequencing data are available in the European Nucleotide Archive server under study accession number PRJEB33641.

## References

[1] Moyer V, Freese DK, Whitington PF, Olson AD, Brewer F, Colletti RB, Heyman MB 2004 Guideline for the evaluation of cholestatic jaundice in infants: recommendations of the North American Society for Pediatric Gastroenterology, Hepatology and Nutrition. J Pediatr Gastroenterol Nutr 39:115–128. doi:10.1097/00005176-200408000-00001. 15269615

[2] Dick MC, Mowat AP 1985 Hepatitis syndrome in infancy—an epidemiological survey with 10 year follow up. Arch Dis Child 60:512–516. doi:10.1136/adc.60.6.512. 3874604 PMC1777358

[3] Fawaz R, Baumann U, Ekong U, Fischler B, Hadzic N, Mack CL, McLin VA, Molleston JP, Neimark E, Ng VL, Karpen SJ 2017 Guideline for the evaluation of cholestatic jaundice in infants: joint recommendations of the North American Society for Pediatric Gastroenterology, Hepatology, and Nutrition and the European Society for Pediatric Gastroenterology, Hepatology, and Nutrition. J Pediatr Gastroenterol Nutr 64:154–168. doi:10.1097/MPG.0000000000001334. 27429428

[4] Karpen SJ 2002 Update on the etiologies and management of neonatal cholestasis. Clin Perinatol 29:159–180. doi:10.1016/S0095-5108(03)00069-1. 11917736

[5] Ananth R 2018 Neonatal cholestasis: a primer of selected etiologies. Pediatr Ann 47:e433–e439. doi:10.3928/19382359-20181018-01. 30423185

[6] Boyer JL 2013 Bile formation and secretion. Compr Physiol 3:1035–1078. doi:10.1002/cphy.c120027. 23897680 PMC4091928

[7] de Aguiar Vallim TQ, Tarling EJ, Edwards PA 2013 Pleiotropic roles of bile acids in metabolism. Cell Metab 17:657–669. doi:10.1016/j.cmet.2013.03.013. 23602448 PMC3654004

[8] Maillette de Buy Wenniger L, Beuers U 2010 Bile salts and cholestasis. Dig Liver Dis 42:409–418. doi:10.1016/j.dld.2010.03.015. 20434968

[9] Chiang JY 2013 Bile acid metabolism and signaling. Compr Physiol 3:1191–1212. doi:10.1002/cphy.c120023. 23897684 PMC4422175

[10] Wahlstrom A, Sayin SI, Marschall HU, Backhed F 2016 Intestinal crosstalk between bile acids and microbiota and its impact on host metabolism. Cell Metab 24:41–50. doi:10.1016/j.cmet.2016.05.005. 27320064

[11] Human Microbiome Project Consortium. 2012 Structure, function and diversity of the healthy human microbiome. Nature 486:207–214. doi:10.1038/nature11234. 22699609 PMC3564958

[12] Ridlon JM, Kang DJ, Hylemon PB, Bajaj JS 2014 Bile acids and the gut microbiome. Curr Opin Gastroenterol 30:332–338. doi:10.1097/MOG.0000000000000057. 24625896 PMC4215539

[13] Sayin SI, Wahlstrom A, Felin J, Jantti S, Marschall HU, Bamberg K, Angelin B, Hyotylainen T, Oresic M, Backhed F 2013 Gut microbiota regulates bile acid metabolism by reducing the levels of tauro-beta-muricholic acid, a naturally occurring FXR antagonist. Cell Metab 17:225–235. doi:10.1016/j.cmet.2013.01.003. 23395169

[14] Begley M, Gahan CG, Hill C 2005 The interaction between bacteria and bile. FEMS Microbiol Rev 29:625–651. doi:10.1016/j.femsre.2004.09.003. 16102595

[15] Ridlon JM, Kang DJ, Hylemon PB 2006 Bile salt biotransformations by human intestinal bacteria. J Lipid Res 47:241–259. doi:10.1194/jlr.R500013-JLR200. 16299351

[16] Inagaki T, Moschetta A, Lee YK, Peng L, Zhao G, Downes M, Yu RT, Shelton JM, Richardson JA, Repa JJ, Mangelsdorf DJ, Kliewer SA 2006 Regulation of antibacterial defense in the small intestine by the nuclear bile acid receptor. Proc Natl Acad Sci U S A 103:3920–3925. doi:10.1073/pnas.0509592103. 16473946 PMC1450165

[17] Boursier J, Mueller O, Barret M, Machado M, Fizanne L, Araujo-Perez F, Guy CD, Seed PC, Rawls JF, David LA, Hunault G, Oberti F, Calès P, Diehl AM 2016 The severity of nonalcoholic fatty liver disease is associated with gut dysbiosis and shift in the metabolic function of the gut microbiota. Hepatology 63:764–775. doi:10.1002/hep.28356. 26600078 PMC4975935

[18] Caussy C, Loomba R 2018 Gut microbiome, microbial metabolites and the development of NAFLD. Nat Rev Gastroenterol Hepatol 15:719–720. doi:10.1038/s41575-018-0058-x. 30158571

[19] Lv LX, Fang DQ, Shi D, Chen DY, Yan R, Zhu YX, Chen YF, Shao L, Guo FF, Wu WR, Li A, Shi HY, Jiang XW, Jiang HY, Xiao YH, Zheng SS, Li LJ 2016 Alterations and correlations of the gut microbiome, metabolism and immunity in patients with primary biliary cirrhosis. Environ Microbiol 18:2272–2286. doi:10.1111/1462-2920.13401. 27243236

[20] Sabino J, Vieira-Silva S, Machiels K, Joossens M, Falony G, Ballet V, Ferrante M, Van Assche G, Van der Merwe S, Vermeire S, Raes J 2016 Primary sclerosing cholangitis is characterised by intestinal dysbiosis independent from IBD. Gut 65:1681–1689. doi:10.1136/gutjnl-2015-311004. 27207975 PMC5036217

[21] Chen Y, Yang F, Lu H, Wang B, Chen Y, Lei D, Wang Y, Zhu B, Li L 2011 Characterization of fecal microbial communities in patients with liver cirrhosis. Hepatology 54:562–572. doi:10.1002/hep.24423. 21574172

[22] Kakiyama G, Pandak WM, Gillevet PM, Hylemon PB, Heuman DM, Daita K, Takei H, Muto A, Nittono H, Ridlon JM, White MB, Noble NA, Monteith P, Fuchs M, Thacker LR, Sikaroodi M, Bajaj JS 2013 Modulation of the fecal bile acid profile by gut microbiota in cirrhosis. J Hepatol 58:949–955. doi:10.1016/j.jhep.2013.01.003. 23333527 PMC3936319

[23] Bajaj JS, Hylemon PB, Ridlon JM, Heuman DM, Daita K, White MB, Monteith P, Noble NA, Sikaroodi M, Gillevet PM 2012 Colonic mucosal microbiome differs from stool microbiome in cirrhosis and hepatic encephalopathy and is linked to cognition and inflammation. Am J Physiol Gastrointest Liver Physiol 303:G675–G685. doi:10.1152/ajpgi.00152.2012. 22821944 PMC3468538

[24] Bajaj JS, Heuman DM, Hylemon PB, Sanyal AJ, White MB, Monteith P, Noble NA, Unser AB, Daita K, Fisher AR, Sikaroodi M, Gillevet PM 2014 Altered profile of human gut microbiome is associated with cirrhosis and its complications. J Hepatol 60:940–947. doi:10.1016/j.jhep.2013.12.019. 24374295 PMC3995845

[25] Qin N, Yang F, Li A, Prifti E, Chen Y, Shao L, Guo J, Le Chatelier E, Yao J, Wu L, Zhou J, Ni S, Liu L, Pons N, Batto JM, Kennedy SP, Leonard P, Yuan C, Ding W, Chen Y, Hu X, Zheng B, Qian G, Xu W, Ehrlich SD, Zheng S, Li L 2014 Alterations of the human gut microbiome in liver cirrhosis. Nature 513:59–64. doi:10.1038/nature13568. 25079328

[26] MahmoudianDehkordi S, Arnold M, Nho K, Ahmad S, Jia W, Xie G, Louie G, Kueider-Paisley A, Moseley MA, Thompson JW, St John Williams L, Tenenbaum JD, Blach C, Baillie R, Han X, Bhattacharyya S, Toledo JB, Schafferer S, Klein S, Koal T, Risacher SL, Kling MA, Motsinger-Reif A, Rotroff DM, Jack J, Hankemeier T, Bennett DA, De Jager PL, Trojanowski JQ, Shaw LM, Weiner MW, Doraiswamy PM, van Duijn CM, Saykin AJ, Kastenmüller G, Kaddurah-Daouk R 2019 Altered bile acid profile associates with cognitive impairment in Alzheimer’s disease—an emerging role for gut microbiome. Alzheimers Dement 15:76–92. doi:10.1016/j.jalz.2018.07.217. 30337151 PMC6487485

[27] Chen H-L, Wu S-H, Hsu S-H, Liou B-Y, Chen H-L, Chang M-H 2018 Jaundice revisited: recent advances in the diagnosis and treatment of inherited cholestatic liver diseases. J Biomed Sci 25:75. doi:10.1186/s12929-018-0475-8. 30367658 PMC6203212

[28] Stelten BML, van de Warrenburg BPC, Wevers RA, Verrips A 2019 Movement disorders in cerebrotendinous xanthomatosis. Parkinsonism Relat Disord 58:12–16. doi:10.1016/j.parkreldis.2018.07.006. 30054180

[29] Macfarlane S, Macfarlane GT 2003 Regulation of short-chain fatty acid production. Proc Nutr Soc 62:67–72. doi:10.1079/PNS2002207. 12740060

[30] Turroni F, Milani C, Duranti S, Mancabelli L, Mangifesta M, Viappiani A, Lugli GA, Ferrario C, Gioiosa L, Ferrarini A, Li J, Palanza P, Delledonne M, van Sinderen D, Ventura M 2016 Deciphering bifidobacterial-mediated metabolic interactions and their impact on gut microbiota by a multi-omics approach. ISME J 10:1656–1668. doi:10.1038/ismej.2015.236. 26859770 PMC4918443

[31] Rao RK, Samak G 2013 Protection and restitution of gut barrier by probiotics: nutritional and clinical implications. Curr Nutr Food Sci 9:99–107. 24353483 10.2174/1573401311309020004 PMC3864899

[32] Miquel S, Martin R, Rossi O, Bermudez-Humaran LG, Chatel JM, Sokol H, Thomas M, Wells JM, Langella P 2013 Faecalibacterium prausnitzii and human intestinal health. Curr Opin Microbiol 16:255–261. doi:10.1016/j.mib.2013.06.003. 23831042

[33] Odenwald MA, Turner JR 2017 The intestinal epithelial barrier: a therapeutic target? Nat Rev Gastroenterol Hepatol 14:9–21. doi:10.1038/nrgastro.2016.169. 27848962 PMC5554468

[34] Fath MJ, Kolter R 1993 ABC transporters: bacterial exporters. Microbiol Rev 57:995–1017. 8302219 10.1128/mr.57.4.995-1017.1993 PMC372944

[35] Xie G, Zhong W, Li H, Li Q, Qiu Y, Zheng X, Chen H, Zhao X, Zhang S, Zhou Z, Zeisel SH, Jia W 2013 Alteration of bile acid metabolism in the rat induced by chronic ethanol consumption. FASEB J 27:3583–3593. doi:10.1096/fj.13-231860. 23709616 PMC3752538

[36] Wang Y, Gao X, Ghozlane A, Hu H, Li X, Xiao Y, Li D, Yu G, Zhang T 2018 Characteristics of faecal microbiota in paediatric Crohn’s disease and their dynamic changes during infliximab therapy. J Crohns Colitis 12:337–346. doi:10.1093/ecco-jcc/jjx153. 29194468

[37] Edgar RC 2010 Search and clustering orders of magnitude faster than BLAST. Bioinformatics 26:2460–2461. doi:10.1093/bioinformatics/btq461. 20709691

[38] Edgar RC 2016 UNOISE2: improved error-correction for Illumina 16S and ITS amplicon sequencing. bioRxiv doi:10.1101/081257.

[39] Zakrzewski M, Proietti C, Ellis JJ, Hasan S, Brion MJ, Berger B, Krause L 2017 Calypso: a user-friendly web-server for mining and visualizing microbiome-environment interactions. Bioinformatics 33:782–783. doi:10.1093/bioinformatics/btw725. 28025202 PMC5408814

[40] Ward T, Larson J, Meulemans J, Hillmann B, Lynch J, Sidiropoulos D, Spear J, Caporaso G, Blekhman R, Knight R, Fink R, Knights D 2017 BugBase predicts organism level microbiome phenotypes. bioRxiv doi:10.1101/133462.

[41] Langille MG, Zaneveld J, Caporaso JG, McDonald D, Knights D, Reyes JA, Clemente JC, Burkepile DE, Vega Thurber RL, Knight R, Beiko RG, Huttenhower C 2013 Predictive functional profiling of microbial communities using 16S rRNA marker gene sequences. Nat Biotechnol 31:814–821. doi:10.1038/nbt.2676. 23975157 PMC3819121

