# Supplementary material for: Gut Microbiota Dysbiosis Is Associated with Altered Bile Acid Metabolism in Infantile Cholestasis
Source: mSystems. 2019 Dec 17;4(6):e00463-19. doi: 10.1128/mSystems.00463-19 (PMC6918028; doi:10.1128/mSystems.00463-19)
Supplement: TABLE S1 [file mSystems.00463-19-st002.docx]

| **Taxa** | **P** | **FDR** | **P adjusted** | **P (Tukeys) HC-IHF** | **P (Tukeys) HC-CJ** | **P (Tukeys) IHF-CJ** | **IHF mean** | **CJ mean** | **HC mean** |
| --- | --- | --- | --- | --- | --- | --- | --- | --- | --- |
| p__Firmicutesc__g__Clostridium_sensu_stricto_OTU2 | 0.0021 | 0.086 | 0.25 | 0.89 | 0.0095 | 0.01 | 0.22 | 7.6 | 1.41 |
| p__Firmicutesc__g__Streptococcus__s__Streptococcus_salivarius_subsp._salivarius_OTU13 | 0.019 | 0.16 | 1 | 0.66 | 0.015 | 0.32 | 3.25 | 6.15 | 1.44 |
| p__Proteobacteria__f__Enterobacteriaceae_OTU24 | 0.00000071 | 0.000087 | 0.000087 | 1 | 0.000004 | 0.00018 | 7.22 | 27.79 | 6.86 |
| p__Firmicutesc__g__Veillonella_OTU40 | 0.0011 | 0.068 | 0.13 | 0.27 | 0.00068 | 0.28 | 2.09 | 3.86 | 0.23 |
| p__Firmicutesc__g__Gemella__s__Gemella_haemolysans_OTU45 | 0.032 | 0.2 | 1 | 0.026 | 0.7 | 0.1 | 0.082 | 0.024 | 0.0044 |
| p__Firmicutesc__g__Clostridium_sensu_stricto__s__Clostridium_butyricum_OTU46 | 0.033 | 0.2 | 1 | 0.81 | 0.029 | 0.29 | 2.01 | 5.62 | 0.47 |
| p__Firmicutesc__g__Enterococcus_OTU68 | 0.018 | 0.16 | 1 | 0.013 | 0.37 | 0.16 | 9.67 | 3.84 | 0.32 |
| p__Firmicutesc__g__Streptococcus_OTU91 | 0.012 | 0.15 | 1 | 0.0087 | 0.26 | 0.17 | 2.69 | 1.41 | 0.48 |
| p__Actinobacteria__g__Eggerthella__s__Eggerthella_sinensis_OTU58 | 0.0073 | 0.1 | 0.85 | 0.2 | 0.0053 | 0.68 | 0.017 | 0.00054 | 0.052 |
| p__Firmicutesc__g__Lachnospiracea_incertae_sedis__s__Ruminococcus_gnavus_OTU84 | 0.049 | 0.24 | 1 | 0.42 | 0.038 | 0.73 | 0.73 | 0.15 | 1.72 |
| p__Firmicutesc__g__Coprococcus__s__Coprococcus_comes_OTU90 | 0.0075 | 0.1 | 0.86 | 0.15 | 0.0058 | 0.77 | 0.07 | 0.00054 | 0.26 |
| p__Actinobacteria__g__Bifidobacterium_OTU96 | 0.0032 | 0.098 | 0.38 | 1 | 0.0064 | 0.03 | 4.39 | 0.9 | 4.41 |
| p__Firmicutesc__g__Blautia__s__Blautia_wexlerae_OTU102 | 0.014 | 0.16 | 1 | 0.97 | 0.019 | 0.1 | 0.28 | 0.0023 | 0.31 |
| p__Firmicutesc__g__Flavonifractor__s__Flavonifractor_plautii_OTU115 | 0.024 | 0.18 | 1 | 0.7 | 0.02 | 0.33 | 0.36 | 0.00089 | 0.56 |
| p__Bacteroidetes__g__Bacteroides__s__Bacteroides_dorei_OTU118 | 0.02 | 0.16 | 1 | 0.069 | 0.032 | 0.98 | 0.46 | 0.9 | 5.71 |
| p__Firmicutesc__g__Faecalibacterium__s__Faecalibacterium_prausnitzii_OTU151 | 0.0053 | 0.1 | 0.63 | 0.039 | 0.0074 | 1 | 0.061 | 0.073 | 3 |
| p__Bacteroidetes__g__Bacteroides__s__Bacteroides_fragilis_OTU162 | 0.048 | 0.24 | 1 | 0.2 | 0.049 | 0.98 | 1.65 | 1.26 | 5.11 |

**TABLE S1** OTUs with significantly different abundance among cholestatic jaundice (CJ), impaired hepatic function (IHF), and health control (HC) groups.
