# Supplementary figures and images for: Gut Microbiota Dysbiosis Is Associated with Altered Bile Acid Metabolism in Infantile Cholestasis
Source: mSystems. 2019 Dec 17;4(6):e00463-19. doi: 10.1128/mSystems.00463-19 (PMC6918028; doi:10.1128/mSystems.00463-19)

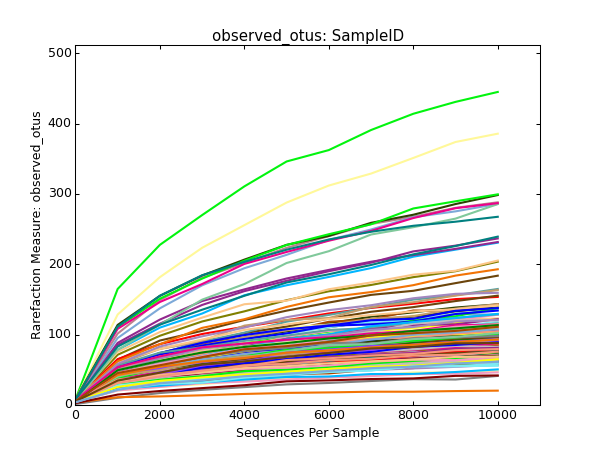

Supplement: FIG S1 [file mSystems.00463-19-sf001.tif]
